# Ventral–Dorsal Subregions in the Posterior Cingulate Cortex Represent Pay and Interest, Two Key Attributes of Job Value

**DOI:** 10.1093/texcom/tgab018

**Published:** 2021-03-09

**Authors:** Shunsui Matsuura, Shinsuke Suzuki, Kosuke Motoki, Shohei Yamazaki, Ryuta Kawashima, Motoaki Sugiura

**Affiliations:** 1 Institute of Development, Aging and Cancer, Tohoku University, Sendai 980-8575, Japan; 2 Frontier Research Institute for Interdisciplinary Sciences, Tohoku University, Sendai 980-0845, Japan; 3 Brain, Mind and Markets Laboratory, Department of Finance, Faculty of Business and Economics, The University of Melbourne, Carlton, VIC 3053, Australia; 4 Department of Food Management, Miyagi University, Sendai 982-0215, Japan; 5 International Research Institute of Disaster Science, Tohoku University, Sendai 980-8572, Japan

**Keywords:** career choice, future simulation, multi-attribute decision-making, posteromedial cortex, precuneus

## Abstract

Career choices affect not only our financial status but also our future well-being. When making these choices, individuals evaluate their willingness to obtain a job (i.e., job values), primarily driven by simulation of future pay and interest. Despite the importance of these decisions, their underlying neural mechanisms remain unclear. In this study, we examined the neural representation of pay and interest. Forty students were presented with 80 job names and asked to evaluate their job values while undergoing functional magnetic resonance imaging (fMRI). Following fMRI, participants rated the jobs in terms of pay and interest. The fMRI data revealed that the ventromedial prefrontal cortex (vmPFC) was associated with job value representation, and the ventral and dorsal regions of the posterior cingulate cortex (PCC) were associated with pay and interest representations, respectively. These findings suggest that the neural computations underlying job valuation conform to a multi-attribute decision-making framework, with overall value signals represented in the vmPFC and the attribute values (i.e., pay and interest) represented in specific regions outside the vmPFC, in the PCC. Furthermore, anatomically distinct representations of pay and interest in the PCC may reflect the differing roles of the two subregions in future simulations.

## Introduction

As one’s job is a key factor in one’s happiness (Fisher 2010), career choices affect not only our financial status but also our future well-being (Diener and Seligman 2004; Bowling et al. 2010; Erdogan et al. 2012). In fact, inappropriate career choices negatively affect mental health (Faragher et al. 2005; Westgaard and Winkel 2011) as well as physical health (Faragher et al. 2005). Given the implications for social and medical policy making, career choices are receiving increasing attention in many fields such as economics, management, and psychology (Cubas and Silos 2017; Wang and Wanberg 2017).

In the fields of psychology and economics, the psychological process of career choice is modeled as a type of multi-attribute decision-making. Multi-attribute decisions are decisions in which options are evaluated according to several attributes (Lancaster 1966; Bettman et al. 1998; Padoa-Schioppa 2011; Busemeyer et al. 2019). Accumulating evidence shows that, for multi-attribute decision-making, the overall value of an option is computed by the weighted addition of attribute values (Bettman et al. 1998; Padoa-Schioppa 2011; Vlaev et al. 2011). On the other hand, several studies have suggested that an individual takes into account multiple attributes of a job when making a career choice (Montgomery and Ramus 2011; Grund 2013). In particular, evaluation of a job is driven by its pay and its interest rather than other attributes (e.g., interpersonal relationships), as shown in a classical international survey (MOW International Research Team 1987). This designation of two key attributes of job value (pay and interest) is consistent with two recent large-scale studies (Skalli et al. 2008; Grund 2013).

In the field of neuroeconomics, the neural mechanisms of multi-attribute decision-making are gradually being revealed. The overall value of an option is independent of sensory modality. Both the ventromedial prefrontal cortex (vmPFC) and striatum, which are components of the dopaminergic reward pathway (Arias-Carrión et al. 2010), represent modality-independent values (Bartra et al. 2013; Clithero and Rangel 2013). In multi-attribute decision-making, the vmPFC is typically assumed to be the region that integrates various attribute values and then computes the overall value of an object (Lim et al. 2013; Suzuki et al. 2017; Busemeyer et al. 2019). On the other hand, recent studies showed that specific brain regions other than the two modality-independent regions can represent individual attribute values. For example, the visual value of a T-shirt (i.e., how much an individual likes its visual features) and its semantic value (i.e., how much an individual likes the meaning and concepts associated with the words printed on the T-shirt) are processed by the fusiform gyrus and posterior superior temporal gyrus, respectively (Lim et al. 2013). Furthermore, Suzuki et al. (2017) suggested that the nutritive attribute values of food (i.e., protein, fat, carbohydrates, and vitamins) are represented in different regions in the lateral orbitofrontal cortex.

Previous neuroeconomic studies, however, did not address the multi-attribute decisions that require future simulation, such as career choice. The decisions examined in previous studies seemed to have involved an immediate reward (i.e., decisions with immediate consequences). The studies did not assume that participants evaluated future values, and their tasks were performed under the assumption that any value would be obtained immediately. On the other hand, career choices require future simulation. As part of a decision-making process, people evaluate a job that they may have in the future. Furthermore, people typically stay at a chosen job for a long time (Oyer 2006; Kahn 2010). Therefore, before a decision is made, future simulation in which the job value is evaluated is required (i.e., decisions with future consequences) (Boyer 2008). Accordingly, the exploration of the neural mechanisms underlying career choice represents an initial attempt to address multi-attribute decision-making with future simulation.

The values of the two key attributes of a job, pay and interest, may be represented in distinct cortical areas. Although both values are likely constructed by imagining the future consequences of the job choice, the underlying simulation processes seem qualitatively different due to the different natures of the values. Typically, the pay is externally given as an outcome of the work (Carroll and Summers 1991; Ryan and Deci 2000; Attanasio and Weber 2010), whereas the interest is internally attained during the process of doing the work (Ryan and Deci 2000; Silvia 2008).

A candidate cortical representation of the two attribute values is in the posterior cingulate cortex (PCC). The PCC has been implicated in various functions potentially useful in guiding the computation of values (Clithero and Rangel 2013). This region is also involved in envisioning the future (future simulation) (Stawarczyk and D’Argembeau 2015; Schacter et al. 2017). Furthermore, segregated subregions in the PCC may represent pay and interest. For example, some studies have suggested that anterior–posterior segregation corresponds to differentially directed thoughts (internally/externally directed thoughts) (Leech et al. 2011), and others have suggested that ventral–dorsal segregation corresponds to the levels of future simulation (outcome/process simulation) (Stawarczyk and D’Argembeau 2015).

In this study, to elucidate the neural mechanism of multi-attribute decisions with future consequences, we examined neural representations of pay and interest, expecting representation of these attribute values in the PCC. Eighty job names were presented, and participants evaluated their willingness to obtain each job (i.e., job value) while their brain activity was measured. Following the evaluation and outside the MRI scanner, participants evaluated the pay and interest of the 80 jobs. To dissociate the subregions representing pay versus interest, the jobs used in this experiment were selected such that pay and interest were independent.

## Materials and Methods

### Participants

All participants provided written informed consent before inclusion in the study, in accordance with the Declaration of Helsinki. The Tohoku University School of Medicine Ethics Committee approved the study protocol (2017-1-220). A total of 40 healthy right-handed students from Tohoku University (21 males; mean age 21.28 [range 18–24] years) participated. Students with majors in medical science or nursing science were excluded as they are typically assumed to become medical doctors and nurses, respectively (Newton and Grayson 2003; Van Iersel et al. 2016), and may therefore have already decided on a job, making them unable to seriously engage in the job valuation task. Handedness was evaluated using the Edinburgh Handedness Inventory (Oldfield 1971). All participants had normal or corrected-to-normal vision and no history of neurological or psychiatric illness. The data from one participant were excluded due to technical errors. The 39 remaining participants (21 males; mean age, 21.33 years; range, 18–24 years) were included in the study.

### Stimuli

To select 80 jobs with minimal correlation between pay and interest, we conducted a preliminary experiment. A total of 349 jobs were chosen from a list of jobs on a job information website (http://careergarden.jp/). Seventeen participants (healthy university students who did not participate in the functional magnetic resonance imaging (fMRI) experiment; 8 males; mean age 22.24 years) rated the pay and interest of the included jobs. The instructions for rating were “Is the job’s pay level high?” and “Is the job interesting? (is this a job you really like?)”; participants rated pay and interest on a Likert-like scale from 1 (no) to 5 (yes). We selected the jobs for which the correlation between pay and interest was the lowest by performing 1 million permutations using R software (version 3.3.3, https://www.r-project.org; R Foundation for Statistical Computing, Vienna, Austria). The list of jobs and the references for their pay are shown in Supplementary Table 1 and Supplementary Table 1.

### Valuation Task (Inside the MRI Scanner)

**Figure 1:**
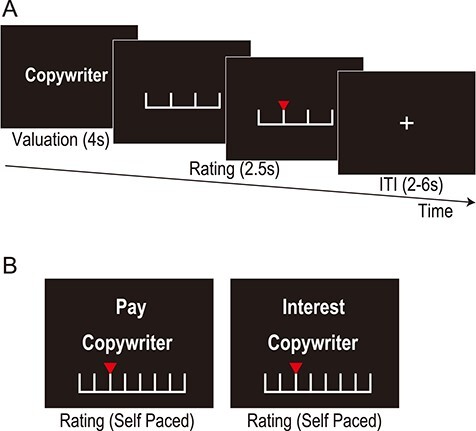
Experimental task. (*A*) Timeline of one trial of the valuation task (fMRI data measured). In each trial, participants viewed a job and evaluated the willingness to obtain the job (i.e., job value), which was rated on a Likert-like scale ranging from 1 (no) to 4 (yes). The trials were separated by a variable intertrial interval phase that lasted between 2 and 6 s. (*B*) Examples of stimuli in trials of the attribute-rating task (outside the MRI scanner). Runs of pay rating and interest rating were divided. The instructions, job name, and rating scale are displayed in the upper, middle, and lower rows, respectively. The same jobs presented during the valuation task were presented one by one, and participants rated the pay of, or interest in, the job on a Likert-like scale ranging from 1 (no) to 8 (yes), with no time constraint.

Each trial was composed of a valuation phase (4 s) and a rating phase (2.5 s). In the valuation phase, one job was presented, and the participants rated their willingness to obtain the job (Fig. 1*A*). Participants were instructed to focus on the extent to which they wanted the job without considering realistic constraints, such as their ability or opportunity. In the rating phase, a Likert-like scale from 1 (no) to 4 (yes) was presented, and the participant rated the job by pressing the key on an MRI-compatible response box attached to their right hand. To exclude motor-related responses of no interest, the response button mapping (left to right and right to left) was randomized across trials. To reduce mistakes, immediate feedback was provided regarding the rating chosen, and participants were allowed to correct it until the end of the rating phase. A white fixation cross was presented between trials (intertrial interval phase, randomly jittered between 2 and 6 s). The task consisted of four runs of 80 trials. In each of the runs, 80 jobs were presented once in random order, and the participants evaluated each job four times in total. We used the average rating over the four trials as the job value. The job names were projected onto a semilucent screen behind the head coil and shown via a mirror attached to the head coil at a visual angle of less than 5°.

### Attribute-rating Task (Outside the MRI Scanner)

Participants rated the pay and interest of the 80 jobs (Fig. 1*B*) by responding to two questions: “Is the job’s pay level high?” and “Is the job interesting? (Is this a job you really like?).” Participants rated pay and interest on a Likert-like scale from 1 (no) to 8 (yes). The instructions used were as described in a previous study (MOW International Research Team 1987). The ratings of pay and interest were divided into different runs, and the order of these runs was randomized across participants. In each run, the order of the jobs was randomized across the job set. The task was given after completing the valuation task so that the participants were not made explicitly aware of these attributes while rating job value.

### Behavioral Data Analysis

We set out to establish whether the willingness to obtain a job (i.e., job value) is guided by its pay and interest.

First, we inspected rating histograms of job value, pay, and interest (Supplementary Table 1 and Supplementary Fig. 1) and confirmed that pay and interest were almost independent; although significantly positive, the mean of correlations between the two attributes calculated at the individual level was small [mean ± standard deviation (SD) = 0.16 ± 0.21, *t* (38) = 4.69, *P* < 0.001]. Furthermore, we confirmed intersession consistency among the four sessions for rating of job value by calculating the Fleiss kappa (κ) (Fleiss 1971) value at the individual level using R software. The median κ value across participants was 0.61 (25% quartile: 0.51; 75% quartile: 0.74), indicating at least moderate or substantial intersession consistency (Landis and Koch 1977).

In the main analysis, we employed generalized linear mixed models (GLMMs; Baayen et al. 2008) implemented in MATLAB (MathWorks) to confirm that the two-attribute-value model allowed better prediction of job value compared to reduced-attribute-value models (i.e., pay-only model, interest-only model, and no-attribute-value model). In all models, the dependent variable was the job value, which was modeled assuming a normal distribution. The independent variables were the attribute values in each model; the variables were pay and interest in the two-attribute-value model, pay in the pay-only model, interest in the interest-only model, and no attribute value was applied in the no-attribute-value model. The model specifications in Wilkinson notation were as follows: two-attribute-value model, job value ~ 1 + pay + interest + (1 + pay + interest | subject); pay-only model, job value ~ 1 + pay + (1 + pay | subject); interest-only model, job value ~ 1 + interest + (1 + interest | subject); no-attribute-value model, job value ~ 1 + (1 | subject)]. The magnitudes of pay and interest were their rating scores in an attribute-rating task. We used Akaike information criterion (AIC) (Akaike 1998) values to compare the predictions of the models.

### Image Acquisition and Data Analysis

#### MRI Data Acquisition

All MRI data were acquired using a 3 T Philips Achieva scanner (Philips, Amsterdam, Netherlands). Functional images were acquired using echo-planar functional images sensitive to blood oxygenation level-dependent contrast (64 × 64 matrix, repetition time = 2750 ms, echo time = 30 ms, flip angle = 80°, field of view = 192 mm, 44 slices, 3-mm slice thickness). In each session, initial dummy volumes were excluded for magnetic stabilization. In total, the remaining 1140 volumes used in the analysis were acquired in 52.25 min.

#### fMRI Data Processing

Analyses of the fMRI data were performed using Statistical Parametric Mapping (SPM12; Wellcome Department of Cognitive Neurology) implemented in MATLAB (MathWorks). Regions of interest (ROIs) were created using the SPM toolbox MarsBaR (http://marsbar.sourceforge.net). All of the images from each participant were preprocessed using the following procedure: corrections for slice timing and head motion, spatial normalization to the echo-planar imaging Montreal Neurological Institute (MNI) template, and smoothing using a Gaussian kernel of 8-mm full width at half maximum.

#### fMRI Data Analysis

Statistical analyses were performed using a conventional two-level approach for multiple-subject fMRI datasets. At the within-subject level, trial-related activity was estimated within the framework of a general linear model (GLM). Next, at the group level, the activation profiles of the identified brain regions were examined by one-sample *t* test. We performed ROI analysis for further quantitative assessment of the functional segregation of the activation clusters representing pay and interest.

At the within-subject level, we estimated GLMs to identify the brain regions representing pay, interest, and job value. The activity was modeled by convolving a time-series model of the neural response with a canonical hemodynamic response function. A first-degree autoregressive [AR(1)] model was used to correct for temporal autocorrelation. We modeled a regressor for activation during job presentation and rating scale presentation, and additional regressors for parametric modulation of activation during job presentation by the values (i.e., job value, pay, and interest). As the job value depends on pay and interest, the regression of three variables in a single model at the same time causes multiple collinearity. Therefore, to solve this problem, we used two separate GLMs for job value and the two attribute values. For GLM1, job value was determined as the average rating of the scores entered during presentation of the rating scale across the four sessions. For GLM2, the values of pay and interest were obtained in an attribute-rating task; we did not use orthogonalization of regressors. For both GLMs, regressors for six parameters of head motion were included as covariates. High-pass filtering with a frequency cut-off of 1 cycle/128 s was applied to decrease the effects of low-frequency noise.

At the group level, to identify the brain regions indicating significantly positive effects of job value (i.e., from GLM1) and the two attribute values (i.e., pay and interest; from GLM2), we conducted one-sample *t* tests of the coefficients for these three variables across all participants. The statistical threshold at each voxel was *P* < 0.001 (uncorrected), which was corrected to the family-wise error of *P* < 0.05 for multiple comparisons at the cluster level.

As group-level results indicated that activation clusters representing pay and interest were located somewhat more ventrally and dorsally in the PCC, respectively (see Results for details), we performed a quantitative analysis of spatial segregation within the PCC. We tested for spatial segregation according to pay and interest along the ventral–dorsal midline axis within the PCC using an approach similar to those described in previous studies (Nicolle et al. 2012; Sul et al. 2015). First, we obtained a sagittal view of the statistical parametric map representing both pay and interest (map of average contrast of pay and interest with a threshold of *P* < 0.001, uncorrected). Second, we generated four ROIs with equally spaced coordinates spanning the ventral-to-dorsal extent of the activation cluster (spheres of radius 4 mm with the following central coordinates: ROI 1, *x* = 0, *y* = −57, *z* = 18; ROI 2, *x* = 0, *y* = −62, *z* = 25; ROI 3, *x* = 0, *y* = −67, *z* = 32; ROI 4, *x* = 0, *y* = −72, *z* = 39; Fig. 4*A*). The number of ROIs was set as four, which was the maximum number that could be placed without overlaps between neighboring ROIs. Using GLM2, the coefficient of neural activation associated with pay and that with interest at the time of job presentation were extracted from the ROIs for each individual. Finally, we ran a GLMM for spatial segregation (i.e., GLMM1). The dependent variable was the coefficient, which was modeled assuming a normal distribution. The independent variables were type of attribute value (pay: 0, interest: 1), ROI location (ROI 1: 1, ROI 2: 2, ROI 3: 3, ROI 4: 4), and the interaction between type of attribute value and ROI location. The model specification in Wilkinson notation was as follows: the coefficient ~1 + type of attribute value * ROI location + (1 + type of attribute value * ROI location | subject). We expected the GLMM results to show the interaction as a fixed effect to reveal segregated representation of pay and interest in the ventral and dorsal PCC. Furthermore, we additionally constructed two GLMMs to confirm the spatial gradient of neural activation associated with each attribute value—the first was a GLMM for determining the spatial gradient of neural activation associated with pay (i.e., GLMM2) and the second was a GLMM for determining the spatial gradient of neural activation associated with interest (i.e., GLMM3). The dependent variable was the coefficient of neural activation associated with the model’s attribute value, that is, the variable was the coefficient of neural activation associated with pay in GLMM2 and that associated with interest in GLMM3. The independent variable was ROI location. The model specifications in Wilkinson notation were as follows: GLMM2, coefficient of neural activation associated with pay ~ 1 + ROI location + (1 + ROI location | subject); GLMM3, coefficient of neural activation associated with interest ~ 1 + ROI location + (1 + ROI location | subject).

## Results

### Behavioral Data

The two-attribute-value model was better at predicting job value compared to the reduced-attribute-value models. The AIC value of the two-attribute-value model (6319.0) was lower than those of the reduced-attribute-value models (no-attribute-value model: 8165.8; pay-only model: 8025.3; interest-only model: 6375.8). Furthermore, in the two-attribute-value model, the effects of pay and interest on job value were significantly positive [coefficient for pay (${\beta}_{\mathrm{pay}}$) = 0.05, standard error (SE) = 0.01, *t* = 3.93, *P* < 0.001; ${\beta}_{\mathrm{interest}}$ = 0.30, SE = 0.02, *t* = 13.29, *P* < 0.001], validating the contributions of these attribute values to job value.

### Neural Representation of Job and Attribute Values

**Figure 2:**
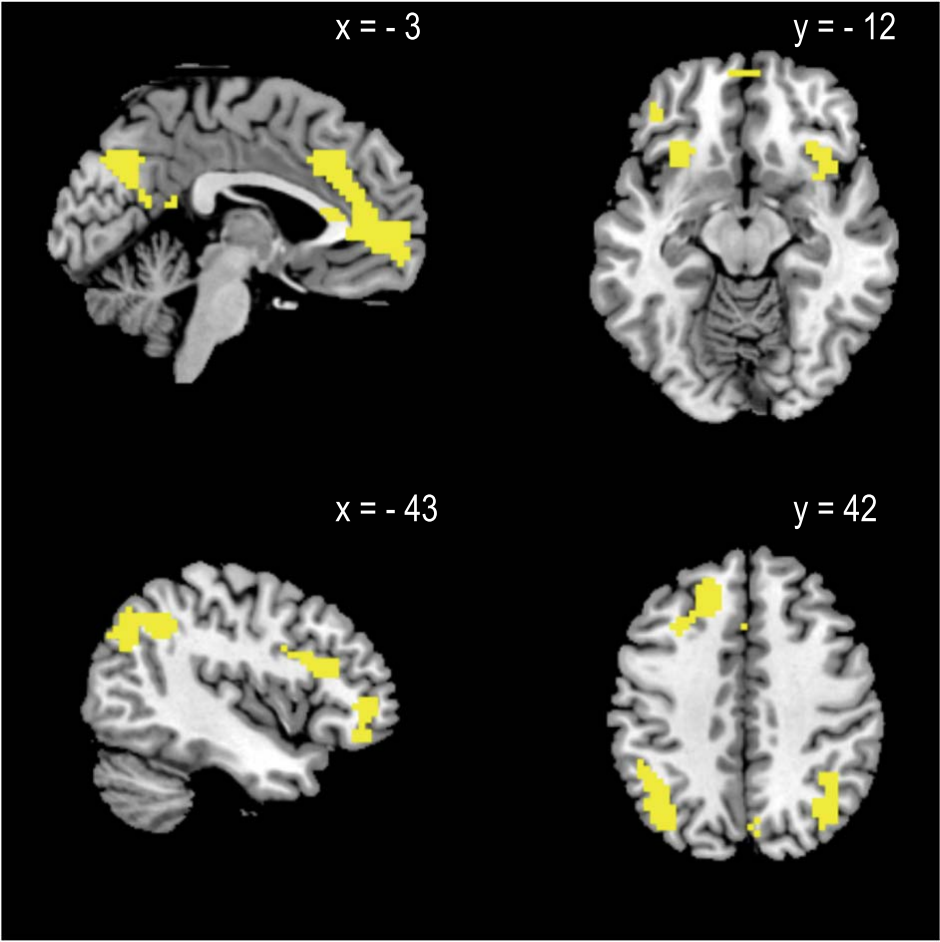
Regions representing job value (effect of job value). The map of activation was overlaid on the Ch2bet template as implemented in MRIcron and shown in sagittal (top left and bottom left) and axial sections (top right and bottom right). The statistical threshold at the voxel level was set at *P* < 0.001 and corrected for multiple comparisons at the cluster level (family-wise error, *P* < 0.05).

We identified the brain regions representing job value and the two attribute values. Significant effects of job value in GLM1 were identified in the vmPFC, PCC, left inferior frontal gyrus, right and left inferior frontal gyrus, left superior frontal gyrus, left inferior parietal lobule, and right angular gyrus (Table 1, Fig. 2).

**Table 1:** Regions representing job and attribute values

Region	MNI coordinates (mm)				*t*	*k*	*P*
	L/R	*x*	*y*	*z*			
**Job value**							
Ventromedial prefrontal cortex	R/L	3	44	10	5.58	489	< 0.001
Posterior cingulate cortex	R	6	−67	37	4.77	191	< 0.001
Inferior frontal gyrus	L	−27	11	−23	5.38	60	0.019
Inferior frontal gyrus	L	−51	23	28	4.89	96	0.002
Inferior frontal gyrus	R	36	23	−8	4.79	60	0.019
Inferior frontal gyrus	L	−39	41	1	4.62	47	0.048
Superior frontal gyrus	L	−18	35	46	5.05	116	0.001
Inferior parietal lobule	L	−36	−55	40	5.17	184	< 0.001
Angular gyrus	R	42	−70	43	4.61	94	0.002
**Pay**							
Posterior cingulate cortex	R/L	3	−58	22	4.84	74	0.008
**Interest**							
Posterior cingulate cortex	L	−6	−67	31	4.54	58	0.021

Notes: The MNI coordinates (*x*, *y*, *z*) of the activation peak, *t*-value, cluster size (*k*: number of voxels; voxel size = 3 × 3 × 3 mm^3^), and *P*-value (corrected for multiple comparisons at the cluster level) for the activation of each effect are presented.

**Figure 3:**
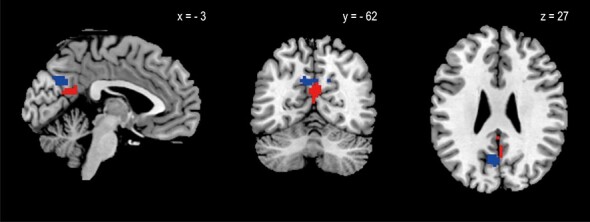
Regions representing pay and interest (effect of pay: red; effect of interest: blue). Sagittal, coronal, and axial sections are shown. The statistical threshold at the voxel level was set to *P* < 0.001 and corrected for multiple comparisons at the cluster level (family-wise error, *P* < 0.05).

In GLM2, both attribute values were represented in the PCC (Fig. 3), whereas the distribution of the activation clusters differed: activation clusters representing pay and interest were located more ventrally and dorsally in the PCC, respectively.

### Spatial Segregation of the PCC for Pay and Interest Representations

**Figure 4:**
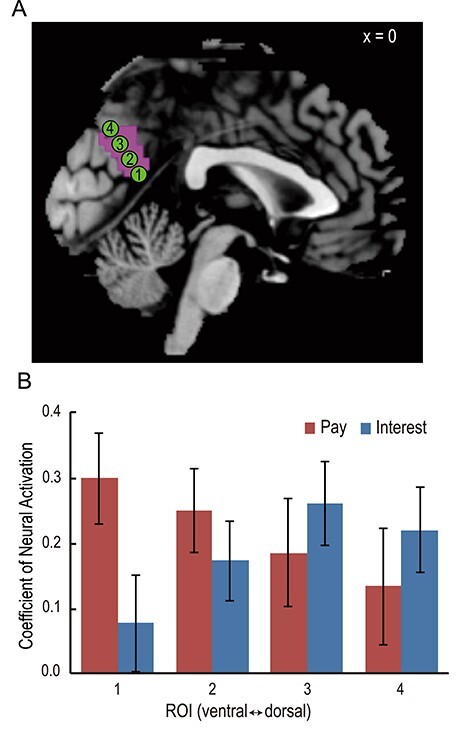
Analysis of spatial gradients for pay and interest. (*A*) The pink region is a sagittal view of the statistical parametric map representing both pay and interest, that is, a map of the average contrast of pay and interest (*P* < 0.001). The green dots represent ROIs. (*B*) Plot of coefficient indicating spatial differences in the effects of pay versus interest. Red and blue bars indicate the mean coefficient of neural activation for pay and interest, respectively. Error bars represent the standard error. “1” represents the ventral end of the ROI and “4” the dorsal end, with the numbers 1–4 in the bar graph corresponding to the ROI numbers in Panel *A*.

We then quantified the spatial segregation according to pay and interest within the PCC. GLMM1 indicated that the interaction between type of attribute value and ROI location was significant (${\beta}_{\mathrm{interaction}}$ = 0.11, SE = 0.04, *t* = 2.54, *P* < 0.05; Fig. 4*B*). Furthermore, GLMM2 indicated that the coefficient of neural activation for pay exhibited a decreasing trend from the ventral to dorsal PCC (${\beta}_{\mathrm{location}}$ = −0.06, SE = 0.03, *t* = −1.76, *P* = 0.08), whereas GLMM3 indicated that the coefficient of neural activation for interest increased significantly from the ventral to dorsal PCC (${\beta}_{\mathrm{location}}$= 0.05, SE = 0.02, *t* = 2.11, *P* < 0.05). These results indicate that the ventral and dorsal regions of the PCC represent pay and interest, respectively.

## Discussion

The present study investigated the cortical representation of pay and interest, two key attributes of job value involved in career decisions. The behavioral data confirmed that pay and interest have positive effects on the willingness to obtain a particular job (i.e., job value), which suggested that our participants were also considering pay and interest when computing job value. fMRI analysis indicated that pay and interest are associated with the PCC. Furthermore, our ROI analysis indicated that segregated subregions of the PCC, the ventral and dorsal regions, are associated with pay and interest, respectively.

These results are consistent with recent neuroscience evidence regarding multi-attribute decision-making. Several studies have revealed that the vmPFC represents integrated values (e.g., Lebreton et al. 2009; Bartra et al. 2013; Clithero and Rangel 2013; Motoki et al. 2019). Moreover, recent studies have revealed that attribute values constituting the value of an item are processed outside the vmPFC and integrated in the vmPFC (Lim et al. 2013; Suzuki et al. 2017; Busemeyer et al. 2019; Suzuki and O’Doherty 2020). The present results revealed that the vmPFC represented job value, whereas the PCC represented attribute values.

The segregated representation of pay and interest between the ventral and dorsal PCC may be explained by the underlying cognitive processes based on the different roles of these subregions in future simulation. These results suggest that the ventral PCC represents pay as the value constructed by imagining what we can obtain when we attain future goals (i.e., outcome simulation) (Boyer 2008). This implication is consistent with previous studies. The ventral PCC has been shown to be involved in the simulation of consumption events (e.g., party and vacation; Peters and Büchel 2010) that individuals typically dream of accessing if sufficient income allowed (Srikant 2013). Furthermore, the ventral end of the PCC, sometimes called the retrosplenial cortex, may be involved in outcome simulation constructed by reward memory (Andrews-Hanna 2012; Ranganath and Ritchey 2012; Andrews-Hanna et al. 2014; Miendlarzewska et al. 2016). On the other hand, the dorsal PCC may be involved in the value constructed by imagining how we can attain future goals (i.e., process simulation) (Pham and Taylor 1999; Taylor and Pham 1999). This implication is consistent with previous studies. For example, one view in psychology suggests that interest comes from performing the job (Ryan and Deci 2000; Silvia 2008), so interest may be involved in process simulation. Previous studies have indicated that process simulation is associated with the dorsal PCC or precuneus (Spreng et al. 2010; Gerlach et al. 2014).

Segregated representation of attribute values in the PCC may be comparable to those in the orbitofrontal cortex in terms of functional organization. In the orbitofrontal cortex, value representation is segregated in an anterior–posterior manner, corresponding to abstraction level differences in sensory information processing: the anterior and dorsal regions correspond to abstract and concrete information processing, respectively (Sescousse et al. 2010; Klein-Flügge et al. 2013; Sescousse et al. 2013; Li et al. 2015). On the other hand, in the PCC, value representation is segregated in a ventral–dorsal manner, corresponding to the different attribute values associated with different simulation levels (i.e., process or outcome) in future simulations.

Unlike other animals, humans are assumed to make sophisticated decisions with future consequences (Seed and Dickerson 2016; Redshaw and Suddendorf 2016). The present study suggests that the ventral and dorsal regions of the PCC represent different attribute values of a job, which may suggest that these regions act together to evaluate future consequences. This view of ventral–dorsal cooperation is consistent with the assumed mechanisms of various future-envisioning processes, such as autobiographical future thought (Spreng et al. 2010; Andrews-Hanna et al. 2014) and episodic future simulation (Benoit and Schacter 2015). Furthermore, the PCC is more developed in modern humans compared with primitive humans and apes (Hagmann et al. 2008; Gunz et al. 2010; Goulas et al. 2014; Bruner et al. 2015; Bruner et al. 2017; Ardesch et al. 2019). This region is involved in unique human functions, such as episodic memory retrieval, self-processing, and visuospatial imagery (Cavanna and Trimble 2006), and it may also be involved in future simulation in humans. Taken together, these findings suggest that career choice may be an example of sophisticated decision-making based on future consequences that is enabled by the integration of multiple attributes processed by highly developed cortical regions in the human brain.

### Limitations

It is important to note that while we showed positive effects of pay and interest on job value, job value is unlikely to depend exclusively on these attributes. Job value also depends on other attributes, such as interpersonal relationships in the workplace (MOW International Research Team 1987). In fact, representation of job value has been identified in various brain regions, such as the left inferior frontal gyrus, right and left inferior frontal gyrus, left superior frontal gyrus, left inferior parietal lobule, and right angular gyrus, in addition to the vmPFC and PCC. These regions may also represent attribute values other than pay and interest. Furthermore, a fruitful research agenda will involve quantifying additional elemental and cultural factors that influence valuation, determining the neural representation of those variables, and establishing how the various signals are integrated to compute an overall value.

Job value measured on a four-point Likert-like scale is related to ordinal utility rather than cardinal utility. Therefore, we cannot argue that the neural representation of that value is associated with reward quantity, in contrast to previous studies using probabilistic reward and willingness-to-pay tasks (Levy and Glimcher 2012). However, we consider ordinal utility sufficient for identifying the regions associated with processing values. In fact, the location of the vmPFC representing the value of an object associated with ordinal utility is the same as that associated with cardinal utility (Levy and Glimcher 2012; Clithero and Rangel 2013), and the location was replicated in the present study.

Interest may be affected by the possibility or plausibility of obtaining a job. People tend to consider plausible actions in simulations of their future (Suddendorf and Corballis 2007; Phillips et al. 2019). Especially, there is evidence that interest is associated with plausibility because they are both associated with similar factors, such as the self-efficacy of the job (Betz 2007) and the abilities needed to do the job (Su et al. 2019). We intended to control for the effect of plausibility by instructing the subjects to focus on the extent to which they wanted the job, but we do not have evidence that this was successful.

Although we were interested in the cognitive processes associated with career choices, we did not assume that the findings presented here can explain all of the processes involved in making a career choice. People typically spend a great deal of time in choosing their actual career, whereas the participants in present study considered the jobs for only a short time. Therefore, it is assumed that there are also a number of neural processes other than those represented in this study.

## Notes

This study was conducted using the MRI scanner and related facilities of the Institute of Development, Aging and Cancer, Tohoku University. The authors thank Ryo Ishibashi, Kentaro Oba, Tetsuya Kageyama, and Kento Takahashi for the data collection. *Conflict of Interest*: None declared.

## Funding

This work was supported by the Japan Society for the Promotion of Science (Grant number KAKENHI 17H06219).

## Supplementary Material

Supplementary_data_tgab018
