# Supplementary material for: Ventral–Dorsal Subregions in the Posterior Cingulate Cortex Represent Pay and Interest, Two Key Attributes of Job Value
Source: Cereb Cortex Commun. 2021 Mar 9;2(2):tgab018. doi: 10.1093/texcom/tgab018 (PMC8152834; doi:10.1093/texcom/tgab018)
Supplement: Supplementary_data_tgab018 [file supplementary_data_tgab018.docx]

**Supplementary data**

| **Table S1.**  List of jobs and references for their pay | |
| --- | --- |
| Job name | Reference for pay |
| Computer graphics designer | 3–5 million yen per year when working for a company |
| Web designer | 3–4 million yen per year |
| Event planner | 3.5–6 million yen per year |
| Wedding planner | 2.5–4.5 million yen per year |
| Photographer | ~3 million yen per year without experience and working for a company |
| Game director | 4–5 million yen per year in their 30s |
| Copywriter | 5–6 million yen per year |
| Chef | 3.6–7 million yen per year |
| Stuntman | 2.5–3 million yen per year when there is a lot of work |
| Scuba diving instructor | 150–180 thousand yen per month from the third year |
| Tennis player | ~500 thousand yen per month when ranked approximately no. 200 in the world |
| Television director | 3–6 million yen per year |
| Pet groomer | 1.8–2.5 million yen per year |
| Dog trainer | 2–3.5 million yen per year |
| Volleyball player | 8–10 million yen per year for a professional player |
| Bartender | 3–4 million yen per year |
| Baker | 150–250 thousand yen per month |
| Piano tuner | ~4 million yen per year |
| Piano teacher | 1.2–1.6 thousand yen per hour when on an outsourcing agreement with a major piano school in an urban area |
| Financial planner | 2–10 million yen per year |
| Fashion designer | 3–4 million yen per year |
| Bridal stylist | 170–190 thousand yen per month as a starting salary |
| Professional boxer | ~0.5–2 million yen per year |
| Professional wrestler | 1–2 million yen per year after the first 5 years |
| Pet sitter | 2–3 million yen per year |
| Bodyguard | 6–8 million yen per year in their 30s |
| Legal licensed condominium manager | 3.3–5.5 million yen per year |
| Make-up artist | 3–4 million yen per year |
| Mental trainer | 3–5 million yen per year when working for a company |
| Yoga instructor | 2.5–4 million yen per year |
| Reporter | > 10 million yen million yen per year with experience and when working for a major broadcast station |
| Road racer | 3–8 million yen per year for top athletes |
| Real estate appraiser | 6–7 million yen per year |
| Junior high school teacher | 7 million yen per year in their 40s |
| Office worker at nursing home | ~2.5 million yen per year |
| Buddhist image maker | 0.3–1 million yen per image |
| Composer | From less than 1 million yen to several tens of millions of yen per year |
| Lyricist | 3–7 million yen per year |
| Adventurer | Income can be earned when a sponsor is found or an offer for adventure-related media is received |
| Labor standards inspector | 5 million yen per year in their 30s |
| Animal breeder | 2–3 million yen per year |
| Maker of Japanese sweets | 3–4 million yen per year as a mid-career employee |
| Librarian of the National Diet Library | 170–180 thousand yen per month as a starting salary |
| Family court probation officer | 4.5 million yen per year at the age of around 30 |
| Novelist | 1–2 million yen per manuscript |
| City council member | ~410 thousand yen per month |
| Registered architect | 4.5–7 million yen per year |
| Sign-language interpreter | ~2.8 million yen per year |
| Japanese language teacher | 3.4–4.2 million yen per year |
| Film promotion | 3.5–6 million yen per year |
| Cook skilled in Japanese cuisine | ~3.3 million yen per year |
| Professional Shogi player | > 10 million yen per year in the top 10^th^ percentile |
| Kabuki actor | ~400 thousand yen per month |
| Dental hygienist | 3.3–3.5 million yen per year |
| Weather forecaster | ~4.5 million yen per year |
| Maritime safety official | ~6.4 million yen per year |
| Tour guide overseas | 2.5–3 million yen per year when working for a company |
| Fire fighter | 6.5–7 million yen per year |
| Registered surveyor | ~4.5 million yen per year |
| Lighting designer | ~4 million yen per year |
| Veterinarian | ~6 million yen per year |
| Physical therapist | 3.5–5 million yen per year |
| Imperial palace guard | ~6.3 million yen per year |
| Nurse | ~4.5 million yen per year |
| Member of a prefectural assembly | ~14 million yen per year |
| Engineer of internal systems | 3–7 million yen per year |
| Secretary | ~4 million yen per year |
| Customs officer | 3.4–7.2 million yen per year |
| Professional keirin cyclist | ~12 million yen per year |
| Music arranger | 2–10 million yen per year |
| Prosthetist | 3–4 million yen per year |
| Playwright | 4–6.5 million yen per year |
| Member of the Self-Defense Forces | ~6.4 million yen per year |
| Ship’s navigating officer | ~7 million yen per year |
| Artist manager | 3–4 million yen per year |
| Licensed cook | ~3.4 million yen per year |
| Locksmith | 3–4 million yen per year |
| Japan Overseas Cooperation Volunteers | 1–2 million yen per year |
| Horse racing jockey | ~10 million yen per year for a jockey of the Japan Association for International Horse Racing |
| High school teacher | ~7.1 million yen per year |

List of 80 jobs used in the present study and references for their pay taken from a job information website (http://careergarden.jp/).

Figure S1. Rating histograms of job value, pay, and interest. The magnitude of job value was the mean of rating scores across the four sessions measured using a Likert-like scale with values ranging from 1 to 4. Pay and interest were measured using a Likert-like scale with values ranging from 1 to 8.
